# *DFNA5* (*GSDME*) c.991-15_991-13delTTC: Founder Mutation or Mutational Hotspot?

**DOI:** 10.3390/ijms21113951

**Published:** 2020-05-31

**Authors:** Kevin T. Booth, Hela Azaiez, Richard J. H. Smith

**Affiliations:** 1 Molecular Otolaryngology and Renal Research Laboratories, Department of Otolaryngology, University of Iowa, Iowa City, IA 52242, USA; kevin_booth@hms.harvard.edu; 2 Department of Neurobiology, Harvard Medical School, Boston, MA 02215, USA

**Keywords:** *DFNA5*, *GSDME*, founder mutation, mutational hotspot, RNA splicing

## Abstract

Deafness due to mutations in the *DFNA5* gene is caused by the aberrant splicing of exon 8, which results in a constitutively active truncated protein. In a large family of European descent (MORL-ADF1) segregating autosomal dominant nonsyndromic hearing loss, we used the OtoSCOPE platform to identify the genetic cause of deafness. After variant filtering and prioritization, the only remaining variant that segregated with the hearing loss in the family was the previously described c.991-15_991-13delTTC mutation in *DFNA5*. This 3-base pair deletion in the polypyrimidine of intron 7 is a founder mutation in the East Asian population. Using ethnicity-informative markers and haplotype reconstruction within the *DFNA5* gene, we confirmed family MORL-ADF1 is of European ancestry, and that the c.991-15_991-13delTTC mutation arose on a unique haplotype, as compared to that of East Asian families segregating this mutation. In-depth audiometric analysis showed no statistical difference between the audiometric profile of family MORL-ADF1 and the East Asian families. Our data suggest the polypyrimidine tract in intron 7 may be a hotspot for mutations.

## 1. Introduction

The dysregulation of RNA-splicing is a common driver of disease. Most commonly, mis-splicing is caused by alterations at the DNA level that impact the binding of one or more of the spliceosome proteins [1]. The resultant mutant mRNA products are targeted for degradation via the nonsense-mediated decay (NMD) pathway, but, in some cases, translation occurs and a mutant protein is produced that drives disease [2,3,4,5]. Such is the case of DFNA5-related hearing loss [6,7].

It is well established that mis-splicing of exon 8 of the *DFNA5 (GSDME)* gene leads to the translation of a mutant protein that causes autosomal dominant (AD) post-lingual progressive nonsyndromic hearing loss (NSHL) [6,7,8,9,10,11,12]. To date, 11 unique mutations at the DNA level have been identified. These mutations are located either in the flanking introns surrounding exon 8, or in exon 8 itself. Although, at the DNA level, all 11 mutations are different, their effects on the mRNA result in translation of the same mutant protein. Functional studies have shown the mutant DFNA5 protein is a component of the apoptotic pathway [13,14].

To date, *DFNA5* mutations have been reported in families of European, Iranian, Chinese, Korean, and Japanese descent [6,7,8,9,10,11,12]. One mutation (c.991-15_991-13delTTC), has been identified in six families of East Asian descent [7,8,9,15,16]. Analysis of flanking single nucleotide polymorphisms in families from Korea, China, and Japan has identified a common haplotype, suggesting that the c.991-15_991-13delTTC mutation is a founder mutation in the East Asian population [8,9]. Here we report the first family of European descent co-segregating post-lingual progressive ADNSHL and the c.991-15_991-13delTTC mutation in *DFNA5*. Haplotype analysis shows this mutation arose on a unique haplotype that differs from the East Asian founder haplotype, suggesting the polypyrimidine tract of intron 7 may be a hotspot for mutations. Audiometric analysis comparing East Asian families carrying the c.991-15_991-13delTTC mutation to the family reported here reveals little racial difference in audioprofiles.

## 2. Results

### 2.1. Clinical Presentation and Audiometric Analysis

**Figure 1:**
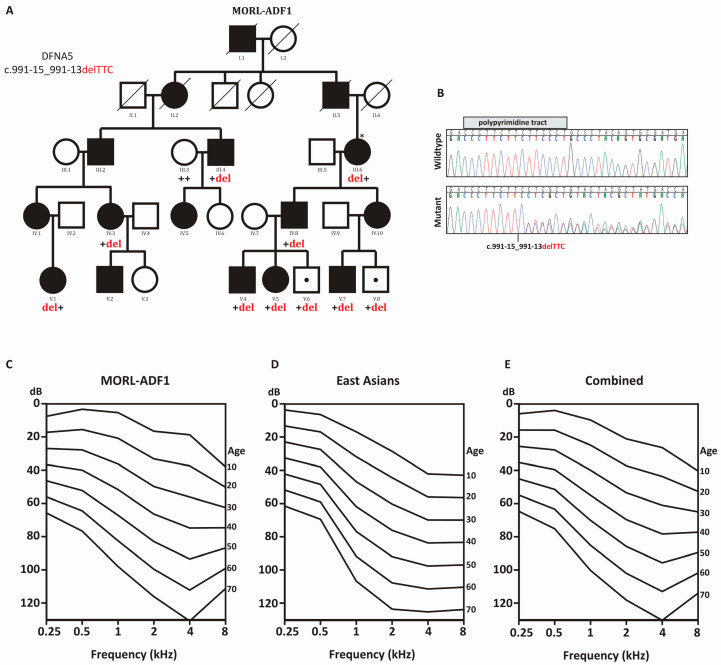
Segregation of the c.991-15_991-13delTTC in *DFNA5* and its related age-related typical audiogram (ARTA). (**A**) Pedigree for family MORL-ADF1. Black symbols are individuals who report hearing loss. Dot symbols represent individuals who carry the *DFNA5* mutation but in whom a formal audiometric evaluation has not been performed and they were too young to display the hearing loss segregating in the extended family. Genotypes of participating family members are shown below, with the c.991-15_991-13delTTC denoted in red. An “*” represents the individual who underwent OtoSCOPE^®^ testing. (**B**) Representative chromatograms from wildtype III.3 (top) and mutant III.6 (bottom) sequences. (**C**–**E**) Age-related typical audiogram (ARTA) for family MORL-AD1, East Asian Families, and combination, respectively. Hearing levels ranged from 0 to 130 dB depending on age and frequency.

The family MORL-ADF1 is a multi-generation kindred of European descent (Figure 1A). Pure tone audiometric evaluation of affected members showed bilateral post-lingual progressive hearing loss that segregated as an autosomal dominant trait. Besides hearing loss, clinical examination of affected individuals was unremarkable.

The hearing loss had a typical onset late in the first decade of life in the high frequencies, with subsequent progression over all frequencies (Table S1). Linear regression analyses of the threshold on age showed an annual threshold deterioration (ATD) of ~0.97 to ~1.9 dB per year (Figure 1B, Figure S1), with the largest progression at 3 kHz. The age-related typical audiogram (ARTA) derived from these data confirmed the down-sloping audiometric configuration, and demonstrated fairly similar progression across all frequencies. Individual III.6 underwent successful cochlear implantation, with good speech recognition and recovery of thresholds back to normal (Figure S2). 

A literature review of other families carrying the c.991-15_991-13delTTC mutation identified 23 audiograms in three families of East Asian descent [7,8,9,15]. Linear regression analysis showed an ATD ranging from ~0.97 to ~1.6 dB per year, with the largest progression at 2 kHz. The ARTA showed progression across all frequencies similar to that seen in family MORL-ADF1 (Figure 1D, Figure S3). 

Progression rates for each frequency (0.25 kHz, 0.5 kHz, 1 kHz, 2 kHz, 4 kHz, and 8 kHz) between family MORL-ADF1 and the East Asian Families showed that the largest difference was at 4 kHz (0.48 dB per year) and the smallest at 0.25 kHz (0.0056 dB per year) (*p* > 0.05; Table 1). There was no significant difference between groups, so we calculated the pooled ATD, which was 0.97 to 1.7 dB per year (Figure 1E, Table 1, and Figure S4).

### 2.2. OtoSCOPE^®^, Segregation Analysis, and Determining Ethnicity

To identify the genetic cause of deafness in family MORL-ADF1, proband III.6 underwent OtoSCOPE^®^ testing. After variant filtering, nine variants remained. Further prioritization for genes associated with ADNSHL and variant classification with the DVD left only the previously described c.991-15_991-13delTTC mutation in *DFNA5* (Figure S5). As part of the OtoSCOPE^®^ design, 55 ancestry-informative single nucleotide polymorphisms (AISNPs) are captured to establish the ethnicity of samples. Analysis of these SNPs defined the ancestry of proband III.6 as a mix of European ethnicities with the highest probability of Toscani, Ashkenazi Jewish, and Hungarian. 

### 2.3. Haplotype Analysis

The c.991-15_991-13delTTC mutation has been linked to a common founder haplotype in East Asians [8,9]. We compared this haplotype to a Korean family we previously published segregating this mutation [7] and to family MORL-ADF1 using data generated by the OtoSCOPE^®^ captured regions. We were able to use 18 SNPs for the *DFNA5* region, nine of which are part of the originally described haplotype. The haplotype for the Korean family matched all nine SNPs; proband III.6 does not share this haplotype (Table 2).

## 3. Discussion

DFNA5 (GDSME) is part of the gasdermin family of proteins that produce pore-forming complexes which lead to pyroptosis (inflammatory cell death). Although DFNA5 is widely expressed throughout the body (https://www.proteinatlas.org/ENSG00000105928-GSDME/tissue), the toxic gain-of-function protein that is produced as a result of skipping exon 8 results only in deafness [6,7,8,9,15,16]. The cause for this limited cochlear phenotype is not known, but may reflect regulatory mechanisms in other tissues, or simply cell turnover. In either case, mutations that result in the skipping of exon 8 cause only post-lingual progressive ADNSHL. 

Proper RNA-splicing requires the ability of spliceosome-associated proteins to recognize and bind their sequence motifs accurately [4,5]. The 11 mutations linked to DFNA5-related deafness disrupt these motifs and result in skipping of exon 8. Using OtoSCOPE^®^, we identified c.991-15_991-13delTTC mutation in *DFNA5*, which segregates with the deafness in the extended family (Figure 1A). This mutation disrupts the polypyrimidine tract at the 3’-end of intron 7. In the East Asian population, it has been shown that this change originated on a founder haplotype shared amongst families from Korea, China, and Japan [8,9]. To exclude East Asian ancestry in Family MORL-ADF1, we utilized 55 AISNPs to show that family MORL-ADF1 is of mixed European ancestry. We also reconstructed a haplotype within the *DFNA5* gene and confirm that MORL-ADF1-III.6 does not carry the same haplotype as families originating from East Asia (Table 2). These data suggest that the polypyrimidine tract preceding exon 8 may be a hotspot for mutations, and that other families of Toscani, Ashkenazi Jewish, and Hungarian heritage may also carry this mutation. Due to the limitations of the SNPs covered by OtoSCOPE^®^ panel, it is not possible to estimate when the mutation occurred. Hotspot mutations in the deafness-associated genes are not uncommon, and have been reported for *KCNQ4* [17], *ACTG1* [18], *WFS1* [19], and *TECTA* [20].

Many of the deafness-associated genes have a genotype-phenotype correlation [21,22], and it has been shown that ethnic background plays a role in hearing thresholds [23]. We compared ATD between family MORL-ADF1 (Figure S2) and a pool of audiograms from East Asian families (Figure S3) carrying the c.991-15_991-13delTTC, and found no age-related difference in thresholds across frequencies (Table 1). This similarity is not surprising, given the mechanism associated with all *DFNA5*-related hearing loss. It is, however, easy to speculate that different mutations in and around exon 8 could have different aberrant splicing efficiencies, and that with “leaky” mutations, the hearing loss leaky might be less severe. Only a large study to compare hearing thresholds and progression rates to mutant RNA levels across all *DFNA5*-linked families will be able to address this possibility.

In conclusion, we report the first non-East Asian family segregating ADNSHL due to the c.991-15_991-13delTTC mutation in *DFNA5*, implicating the polypyrimidine tract in intron 7 as a mutational hotspot. Irrespective of ethnicity, the deafness phenotype caused by the c.991-15_991-13delTTC mutation is similar.

## 4. Materials and Methods

### 4.1. Subjects

A large multi-generation family of European descent (Family MORL-ADF1) was ascertained as part of a genetic study of ADNSHL at the University of Iowa. After obtaining written and informed consent, blood or saliva samples, along with medical information, were collected from participating members. This study was approved by the institutional review boards at the University of Iowa.

### 4.2. Audiometric Profiling

Annual threshold deterioration (ATD) and age-related typical audiograms (ARTA) were derived, as described [22,24,25]. Briefly, standard pure tone audiometry was performed on affected individuals to determine air conduction thresholds from 0.25–8 kHz. After validating binaural symmetry, the binaural mean air conduction threshold (dB Hearing Level, HL) at each frequency was used for further analyses. Next, linear regression analyses of data derived from individual serial audiograms and overall cross-sectional last-visit data were used to evaluate the progression of hearing impairment at individual frequencies. Progression was considered significant if the 95% confidence interval for slope did not include zero for two or more frequencies, and was expressed in dB-per-year. Regression data were used to derive the ARTA, which shows expected thresholds by decade steps in age.

A literature search using the NCBI database PubMed (accessed March 2020) retrieved previously published audiometric data for families segregating the *DFNA5* c.991-15_991-13delTTC mutation. The ATD, progression and ARTA were derived as described above.

### 4.3. OtoSCOPE

Individual III.6 underwent OtoSCOPE^®^ testing, as described [7,26,27]. Briefly, after genomic capture, enriched libraries were pooled and sequenced using an Illumina Hiseq 2000 (Illumina, San Diego, CA, USA). Subsequently, raw reads underwent alignment and variant calling using the Genome Analysis Toolkit (Broad Institute, Cambridge, MA, USA) best practices. After variant calling, variants were annotated using Variant Effect Predictor (VEP), Exome Aggregation Consortium database (ExAC) (http://exac.broadinstitute.org/), the Genome Aggregation Database (gnomAD) (http://gnomad.broadinstitute.org/) and custom annotation from the Deafness Variation Database (DVD) (http://deafnessvariationdatabase.org/) [28]. Next, variants were filtered based on quality and minor allele frequency (MAF). Variants were further prioritized based on predicted functional consequence conservation (phyloP), deleteriousness (Combined Annotation Dependent Depletion (CADD)), and variant classification from the DVD. Copy number variants (CNVs) were elevated using a sliding window method and read depth as described [29].

### 4.4. Segregation Analysis

Sanger sequencing was completed in available family members to confirm segregation of the candidate variant, c.991-15_991-13delTTC, in the *DFNA5* gene (NM_004403) using gene-specific primers flanking exon 8, as described in [7].

### 4.5. Ethnicity-Informative Markers

Fifty-five ancestry inference single nucleotide polymorphisms (AISNPs) from the Forensic Resource/Reference On Genetics—knowledge base (FROG-kb) were used, to ascertain the ethnic background of individual III.6 [30]. Ancestry was determined by calculating the probability and relative likelihoods of ancestry from different reference populations.

### 4.6. Statistical Analysis

Statistical analysis was done using a Student’s *t*-test, with significance considered a *p* < 0.05. 

## Figures and Tables

**Table 1:** Progression rates by frequency.

	0.250 kHz	0.500 kHz	1 kHz	2 kHz	4 kHz	8 kHz
MORL-ADF1	0.9685	1.222	1.543	1.659	1.869	1.221
E. Asians	0.9629	1.050	1.499	1.584	1.385	1.349
Difference	0.0056	0.172	0.044	0.075	0.484	0.128
*p*-value	0.186	0.391	0.691	0.767	0.194	0.829
Combined	0.977	1.186	1.508	1.618	1.733	1.227

The difference represents the absolute value.

**Table 2:** Haplotype analysis for *DFNA5* for the c.991-15_991-13delTTC mutation between East Asians and MORL-ADF1.

SNP	Loc	ADF1-III.6	Korean [7]	Korean [9]	Chinese [9]	Japanese [8]		MAF European (NF)	MAF E. Asian	MAF Global
rs17149912 (T|C)	Ex 9	T|**T**	T|C	T	**C**	**C**	**C**	15.69%	27.68%	19.78%
rs2240005 (G|A)	In 8	G|A	G|**G**	**G**	**G**	**G**	**G**	22.87%	24.33%	30.78%
rs66851582 (C|T)	In 8	C|T	C|**C**	-	-	-	-	14.17%	0.06%	10.88%
rs2074142 (C|T)	In 8	C|**C**	C|**T**	**T**	**T**	**T**	**T**	25.27%	67.16%	34.30%
rs727505273 (GAA|Del)	In 7	GAA|Del	GAA|Del	**Del**	**Del**	**Del**	**Del**	0.00%	0.00%	0.00%
rs17209408 (C|T)	In 7	C|T	C|**C**	**C**	**C**	**C**	**C**	2.80%	0.01%	1.77%
rs141596134 (C|T)	Ex 7	C|**C**	C|**T**	-	-	-	-	0.00%	0.10%	0.01%
rs2721809 (G|A)	In 6	G|**G**	A|**A**	-	-	-	-	42.28%	99.14%	56.34%
rs35529766 (C|Del)	In 6	C|**C**	C|**Del**	**Del**	**Del**	**Del**	**Del**	0.00%	0.00%	0.00%
rs10601416; 35521389 (TA|Del)	In 4	TA|**TA**	Del|**Del**	-	-	-	-	49.08%	99.23%	60.79%
rs876308 (G|A)	In 4	G|A	G|**G**	-	-	-	-	43.60%	99.17%	56.98%
rs876307 (G|T)	In 4	G|**G**	T|**T**	-	-	-	-	42.46%	99.12%	57.01%
rs754553 (C|T)	In 3	C|**C**	C|**T**	**T**	**T**	**T**	**T**	15.04%	45.96%	19.80%
rs2023793 (G|A)	In 3	G|**G**	A|**A**	**A**	**A**	-	-	43.50%	99.23%	56.92%
rs2521768 (C|T)	In 2	C|**C**	T|**T**	**T**	**T**	**T**	**T**	46.55%	79.95%	58.43%
rs150598245 (wt|insGT)	In 2	wt|GT	wt|**GT**	-	-	-	-	1.68%	0.98%	1.30%
rs2521770 (C|T)	In 2	C|T	T|**T**	-	-	-	-	51.88%	99.87%	65.31%
rs768391255 (wt|Ins)	In 2	wt|Ins	-|-	-	-	-	-	16.41%	21.58%	14.58%

Red and bold indicates the c.991-15_991-13delTTC mutation, whereas bold represents morbid haplotype. (-) data not available. MAF from the gnomAD database. Loc: Location. Ex: exon. In: intron.

## Supplementary Materials

Supplementary materials can be found at https://www.mdpi.com/1422-0067/21/11/3951/s1.

## References

[1] Park J.E., Cartegni L. (2017). In Vitro Modulation of Endogenous Alternative Splicing Using Splice-Switching Antisense Oligonucleotides. Methods Mol. Biol..

[2] Hentze M.W., Kulozik A.E. (1999). A perfect message: RNA surveillance and nonsense-mediated decay. Cell.

[3] He F., Jacobson A. (2015). Nonsense-Mediated mRNA Decay: Degradation of Defective Transcripts Is Only Part of the Story. Annu. Rev. Genet..

[4] Matera A.G., Wang Z. (2014). A day in the life of the spliceosome. Nat. Rev. Mol. Cell Biol..

[5] Cooper T.A., Wan L., Dreyfuss G. (2009). RNA and Disease. Cell.

[6] Van Laer L., Huizing E.H., Verstreken M., van Zuijlen D., Wauters J.G., Bossuyt P.J., Van de Heyning P., McGuirt W.T., Smith R.J., Willems P.J. (1998). Nonsyndromic hearing impairment is associated with a mutation in DFNA5. Nat. Genet..

[7] Booth K.T., Azaiez H., Kahrizi K., Wang D., Zhang Y., Frees K., Nishimura C., Najmabadi H., Smith R.J. (2018). Exonic mutations and exon skipping: Lessons learned from DFNA5. Hum. Mutat..

[8] Nishio A., Noguchi Y., Sato T., Naruse T.K., Kimura A., Takagi A., Kitamura K. (2014). A DFNA5 mutation identified in japanese families with autosomal dominant hereditary hearing loss. Ann. Hum. Genet..

[9] Park H.-J., Cho H.-J., Baek J.-I., Ben-Yosef T., Kwon T.-J., Griffith A.J., Kim U.-K. (2010). Evidence for a founder mutation causing DFNA5 hearing loss in East Asians. J. Hum. Genet..

[10] Li-Yang M.-N.N., Shen X.-F.F., Wei Q.-J.J., Yao J., Lu Y.-J.J., Cao X., Xing G.-Q.Q. (2015). IVS8+1 DelG, a Novel Splice Site Mutation Causing DFNA5 Deafness in a Chinese Family. Chin. Med. J. (Engl.).

[11] Chai Y., Chen D., Wang X., Wu H., Yang T. (2014). A novel splice site mutation in DFNA5 causes late-onset progressive non-syndromic hearing loss in a Chinese family. Int. J. Pediatr. Otorhinolaryngol..

[12] Cheng J., Han D.Y., Dai P., Sun H.J., Tao R., Sun Q., Yan D., Qin W., Wang H.Y., Ouyang X.M. (2007). A novel DFNA5 mutation, IVS8+4 A>G, in the splice donor site of intron 8 causes late-onset non-syndromic hearing loss in a Chinese family. Clin. Genet..

[13] Gregan J., Van Laer L., Lieto L.D., Van Camp G., Kearsey S.E. (2003). A yeast model for the study of human DFNA5, a gene mutated in nonsyndromic hearing impairment. Biochim. Biophys. Acta Mol. Basis Dis..

[14] Rogers C., Fernandes-Alnemri T., Mayes L., Alnemri D., Cingolani G., Alnemri E.S. (2017). Cleavage of DFNA5 by caspase-3 during apoptosis mediates progression to secondary necrotic/pyroptotic cell death. Nat. Commun..

[15] Yu C., Meng X., Zhang S., Zhao G., Hu L., Kong X. (2003). A 3-nucleotide deletion in the polypyrimidine tract of intron 7 of the DFNA5 gene causes nonsyndromic hearing impairment in a Chinese family. Genomics.

[16] Wang H., Guan J., Guan L., Yang J., Wu K., Lin Q., Xiong W., Lan L., Zhao C., Xie L. (2018). Further evidence for “gain-of-function” mechanism of DFNA5 related hearing loss. Sci. Rep..

[17] Van Camp G., Coucke P.J., Akita J., Fransen E., Abe S., De Leenheer E.M.R., Huygen P.L.M., Cremers C.W.R.J., Usami S.-I. (2002). A mutational hot spot in theKCNQ4 gene responsible for autosomal dominant hearing impairment. Hum. Mutat..

[18] Wang L., Yan D., Qin L., Li T., Liu H., Li W., Mittal R., Yong F., Chapagain P., Liao S. (2018). Amino acid 118 in the deafness causing (DFNA20/26) ACTG1 gene is a mutational hot spot. Gene Rep..

[19] Choi H.J., Lee J.S., Yu S., Cha D.H., Gee H.Y., Choi J.Y., Lee J.D., Jung J. (2017). Whole-exome sequencing identified a missense mutation in WFS1 causing low-frequency hearing loss: A case report. BMC Med. Genet..

[20] Moteki H., Nishio S., Hashimoto S., Takumi Y., Iwasaki S., Takeichi N., Fukuda S., Usami S. (2012). TECTA mutations in Japanese with mid-frequency hearing loss affected by zona pellucida domain protein secretion. J. Hum. Genet..

[21] Taylor K.R., Booth K.T., Azaiez H., Sloan C.M., Kolbe D.L., Glanz E.N., Shearer A.E., DeLuca A.P., Anand V.N., Hildebrand M.S. (2016). Audioprofile surfaces: The 21st century audiogram. Ann. Otol. Rhinol. Laryngol..

[22] Huygen P.L.M., Pennings R.J.E., Cremers C.W.R.J. (2003). Characterizing and Distinguishing Progressive Phenotypes in Nonsyndromic Autosomal Dominant Hearing Impairment. Audiol. Med..

[23] Daniel W., Kevin B., Azaiez Hela S.R.J. (2020). A Comparative Analysis of Genetic Hearing Loss Phenotypes in European/American and Japanese Populations. Hum. Genet..

[24] Azaiez H., Decker A.R., Booth K.T., Simpson A.C., Shearer A.E., Huygen P.L.M., Bu F., Hildebrand M.S., Ranum P.T., Shibata S.B. (2015). HOMER2, a Stereociliary Scaffolding Protein, Is Essential for Normal Hearing in Humans and Mice. PLoS Genet..

[25] Azaiez H., Booth K.T., Bu F., Huygen P., Shibata S.B., Shearer A.E., Kolbe D., Meyer N., Black-Ziegelbein E.A., Smith R.J.H. (2014). TBC1D24 Mutation Causes Autosomal-Dominant Nonsyndromic Hearing Loss. Hum. Mutat..

[26] Booth K.T., Azaiez H., Kahrizi K., Simpson A.C., Tollefson W.T.A., Sloan C.M., Meyer N.C., Babanejad M., Ardalani F., Arzhangi S. (2015). PDZD7 and hearing loss: More than just a modifier. Am. J. Med. Genet. Part A.

[27] Booth K.T., Kahrizi K., Najmabadi H., Azaiez H., Smith R.J. (2018). Old gene, new phenotype: Splice-altering variants in CEACAM16 cause recessive non-syndromic hearing impairment. J. Med. Genet..

[28] Azaiez H., Booth K.T., Ephraim S.S., Crone B., Black-Ziegelbein E.A., Marini R.J., Shearer A.E., Sloan-Heggen C.M., Kolbe D., Casavant T. (2018). Genomic Landscape and Mutational Signatures of Deafness-Associated Genes. Am. J. Hum. Genet..

[29] Nord A.S., Lee M., King M.-C., Walsh T. (2011). Accurate and exact CNV identification from targeted high-throughput sequence data. Bmc Genom..

[30] Kidd K.K., Speed W.C., Pakstis A.J., Furtado M.R., Fang R., Madbouly A., Maiers M., Middha M., Friedlaender F.R., Kidd J.R. (2014). Progress toward an efficient panel of SNPs for ancestry inference. Forensic. Sci. Int. Genet..

