# Supplementary material for: DFNA5 (GSDME) c.991-15_991-13delTTC: Founder Mutation or Mutational Hotspot?
Source: Int J Mol Sci. 2020 May 31;21(11):3951. doi: 10.3390/ijms21113951 (PMC7312536; doi:10.3390/ijms21113951)
Supplement: Supplementary file 1 [file ijms-21-03951-s001.zip › Supp_Table1.pdf]

Clinical History for family MORL-AD1

Individual	Age of Onset (years)
III.4	<10†
III.6	<10†
IV.3	10-15†
IV.8	10‡
V.1	13‡
V.4	10‡
V.5	NA
V.6	++
V.7	NA
V.8	++

†Self-reported age of onset.
‡Earliest audiogram showing hearing loss.
NA: Not available
++ Individual tested positive for the c.991-15_991-13delTTC mutation but has yet to develop hearing loss.
