# Supplementary figures and images for: DFNA5 (GSDME) c.991-15_991-13delTTC: Founder Mutation or Mutational Hotspot?
Source: Int J Mol Sci. 2020 May 31;21(11):3951. doi: 10.3390/ijms21113951 (PMC7312536; doi:10.3390/ijms21113951)

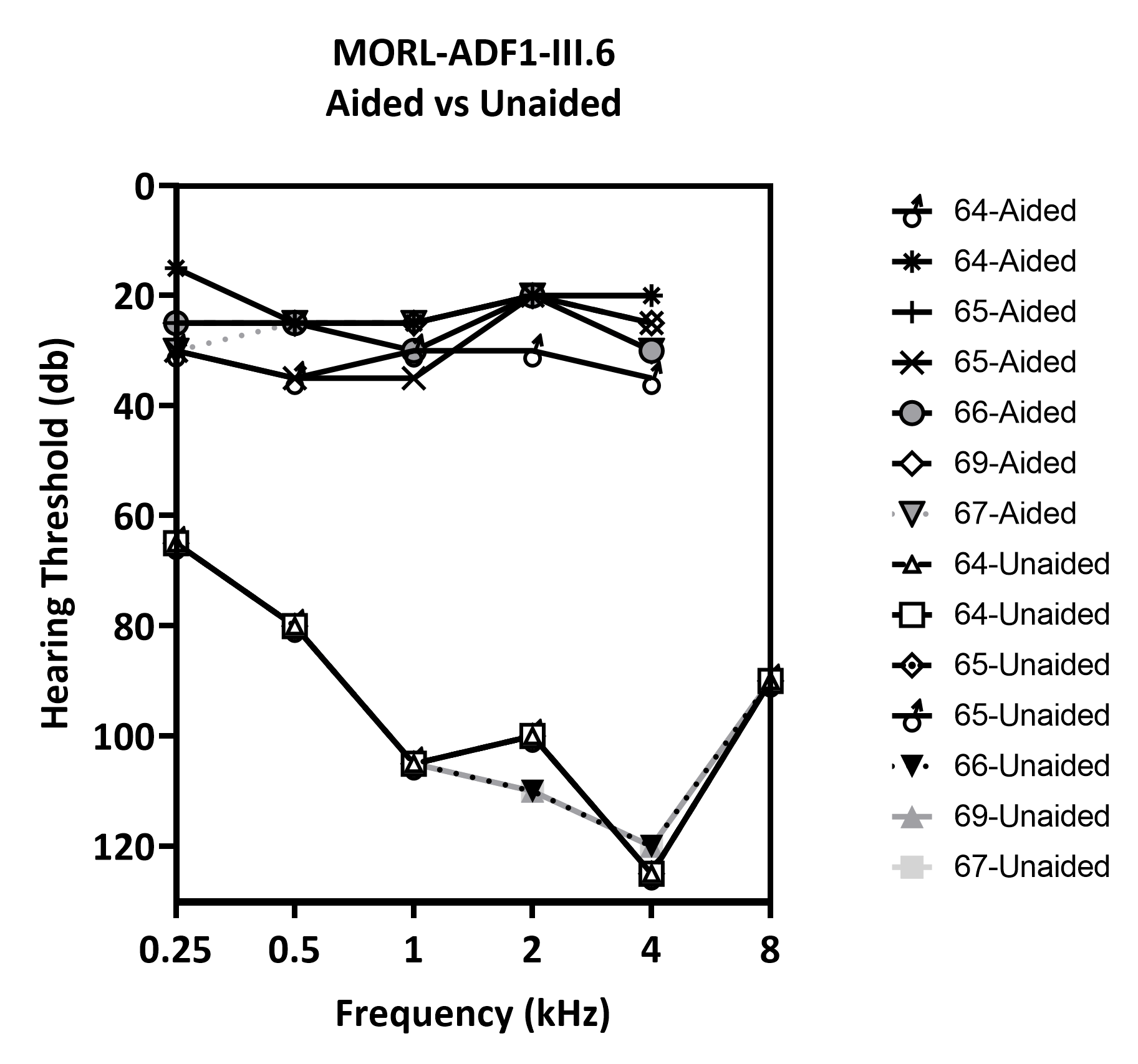

Supplement: Supplementary file 1 [file ijms-21-03951-s001.zip › Supp_Figure2.tif]

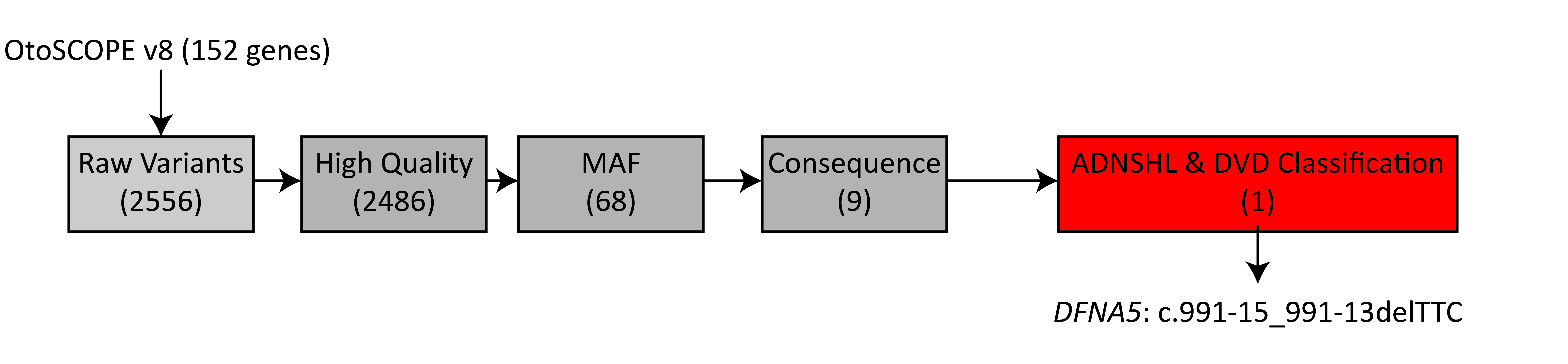

Supplement: Supplementary file 1 [file ijms-21-03951-s001.zip › Supp_Figure5.tif]
